# Microvascular anastomotic coupler for venous end-to-side anastomoses in head and neck reconstructive surgery

**DOI:** 10.1007/s00405-023-08136-0

**Published:** 2023-08-01

**Authors:** Ulrich Kisser, Sophie Koepernik

**Affiliations:** Department of Otorhinolaryngology, Head and Neck Surgery, University of Halle, Ernst-Grube-Strasse 40, 06120 Halle (Saale), Germany

**Keywords:** Free flaps, Reconstructive surgery, Head and neck tumors, Anastomosis, Coupler device

## Abstract

**Background:**

The use of coupler devices has become mainstream in microsurgical end-to-end venous anastomoses (EEA) for free flaps in head and neck reconstruction. Reports about end-to-side venous anastomoses (ESA) using a coupler are scarce, though.

**Methods:**

The surgical technique of end-to-side anastomosis using a coupler device is described. End-to-side anastomoses and end-to-end anastomoses with a vascular coupler are compared with respect to postoperative vascular complications.

**Results:**

124 patients were included, 76 with EEA, 48 with ESA. Postoperative venous complications occurred in 5.3% and 2.1%, respectively.

**Conclusions:**

ESA is a valuable alternative to EEA when using a coupler device providing more flexibility to the surgeon.

**Supplementary Information:**

The online version contains supplementary material available at 10.1007/s00405-023-08136-0.

## Key Points

**Table:** 

MCD are safe
MCD are time-saving
MCD cannot only be used for EEA but also for ESA
The use of MCD helps to overcome vessel size mismatch
Success rates of ESA and EEA are similar when using MCD
Success rates of ESA using MCD are comparable or even superior to those of hand-sewn ESA
The IJV allows for anastomoses of any size and number and at any site along the neck
ESA using MCD is a lot easier if tension of the vessel wall is reduced by gathering the vein
The use of large coupler sizes is associated with lower revision rates
ESA with MCD have a shorter learning curve than hand-sewn ESA

## Background

The use of microvascular coupler devices (MCD) has become mainstream in microsurgical techniques for venous end-to-end anastomoses (EEA) in head and neck reconstruction (HNR) with free flaps. Clinical and experimental data have shown that it is safe, time-saving and has high patency rates when compared to traditional hand-sewn anastomoses [1]. Reports about end-to-side venous anastomoses (ESA) using a coupler are scarce, though.

## Methods

Here, we describe how to use a microvascular coupler device (Synovis Micro Companies Alliance, Birmingham, AL, USA) for end-to-side anastomoses. In a retrospective analysis, ESA and EEA with a vascular coupler are compared with respect to postoperative vascular complications and anastomosis time.

### Surgical anatomy

Free flaps used for reconstruction after head and neck tumor ablation are usually anastomosed to cervical vessels. The branches of the external carotid artery are suited best as donor arteries. Venous anastomoses to the internal jugular vein system have a significantly higher success rate than anastomoses to the external jugular vein (IJV) due to better blood flow and larger calibre [2, 3].

### Surgical technique (Figs. 1, 2, 3, 4, 5 and 6)

**Fig. 1:**
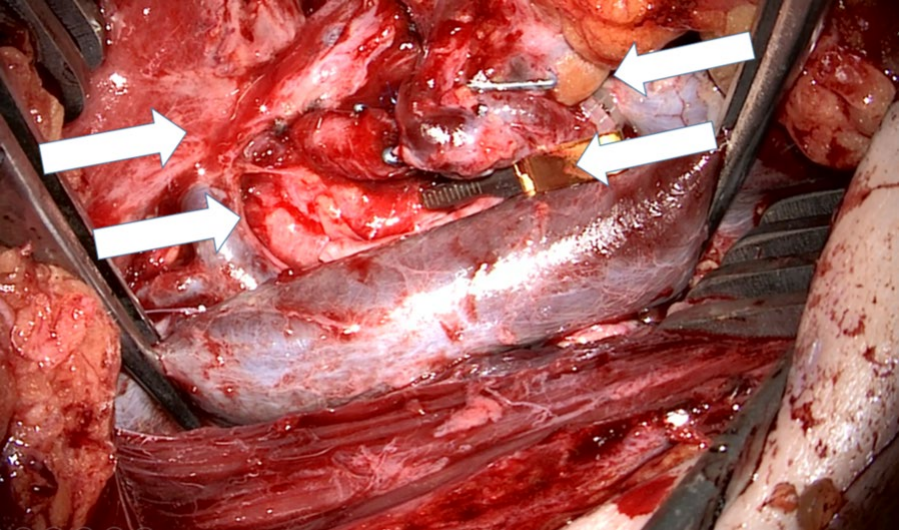
The IJV is shirred by pulling the upper and lower sections of the vein toward each other with two forceps (white arrows)

For ESA the recipient vein, usually, the IJV is dissected 360° over as long a distance as possible. A Satinsky clamp is used to temporarily stop the blood flow. Before the clamp is closed, the vein should be shirred by having an assistant pull the upper and lower sections of the vein toward each other with two forceps (Fig. 1). Otherwise, there is too much tension on the vessel wall when the coupler is to be used. Micro-scissors are used to cut a hole in the vein wall under microscopic control (Fig. 3). The size is adapted to the diameter of the flap vein, which can be measured with a standard vessel-measuring gauge (Fig. 4). Both vessels are then pulled through the coupler rings, everted and mounted on the steel pins using special coupler forceps (Fig. 5) before the rings are approximated and the anastomosis is finished. Other surgical steps have been described elsewhere [4].

**Fig. 2:**
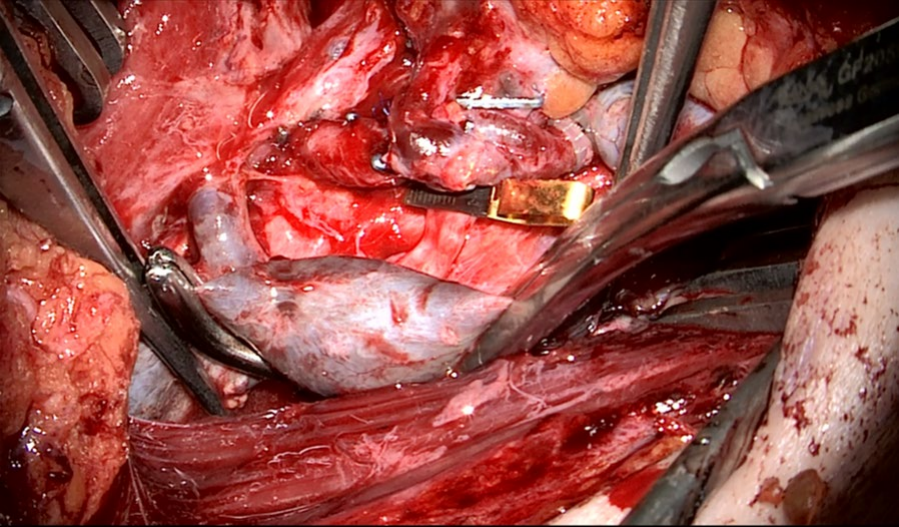
A Satinsky clamp is applied

**Fig. 3:**
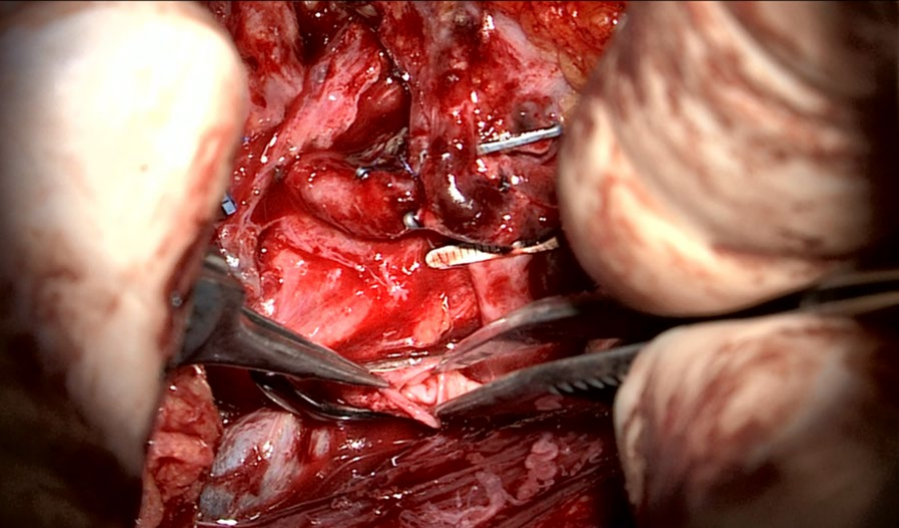
After venotomy

**Fig. 4:**
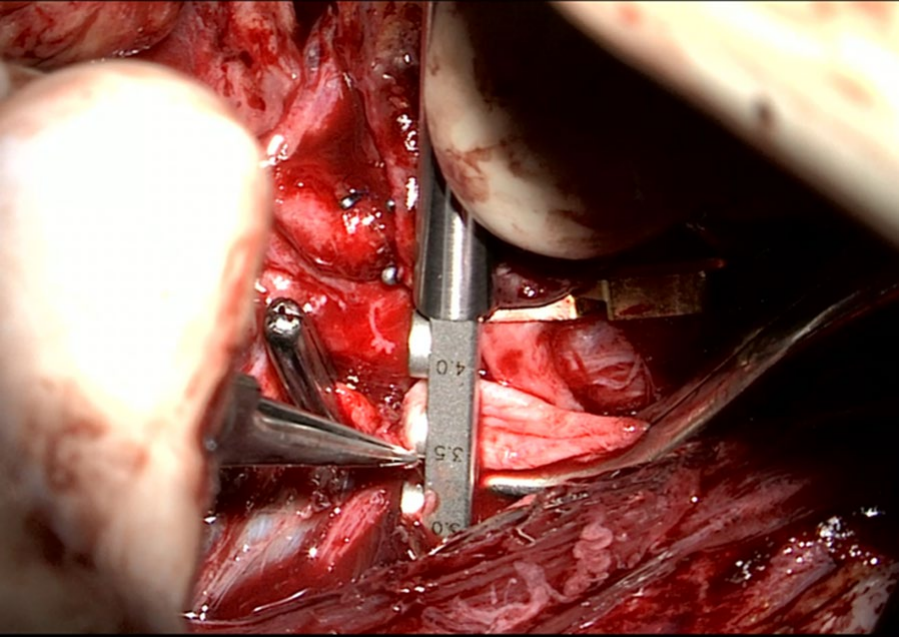
Size of the venotomy is controlled with measuring device

**Fig. 5:**
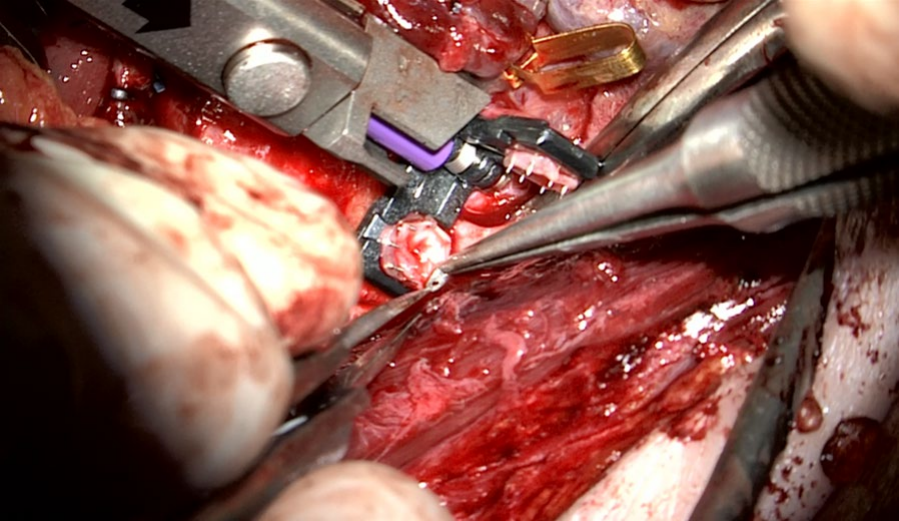
Both vessels are mounted on the steel pins of the coupler rings

**Fig. 6:**
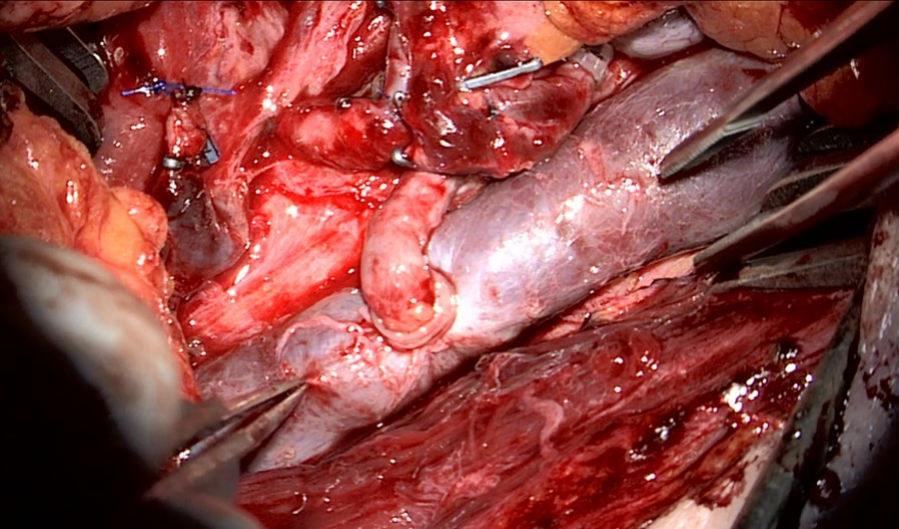
Finished anastomosis

### Indications

Venous ESA can be used as an alternative to EEA in head and neck reconstruction using free flaps. The radial forearm flap is one of the most popular free flaps in head and neck reconstruction. If the superficial venous system (cubital vein) is included when harvesting the flap, large vessel diameters and coupler sizes can be achieved which are associated with lower revision rates [4]. Due to its size the IJV allows for anastomoses of any size. Furthermore, the option of using the IJV for ESA provides more flexibility to the surgeon especially when it comes to patients with difficult anatomy, for example, after radical neck dissection when there are no suitable veins left for EEA.

### Limitations

ESA to the IJV can be performed in most cases, provided the vein can be dissected properly which might be limited after repeated surgical and/or radiation therapy with extraordinary scarring or venous fragility. After radical neck dissection including resection of the IJV, the contralateral IJV can be used.

### How to avoid complications

It is recommended to use the largest possible coupler size [4]. Besides, the same rules and specific perioperative considerations apply as for EEA [2–4].

## Results

124 patients (mean age 62 years, median age 61 years, 92 male) were included. Tumor localisations were oropharynx (*n* = 67), oral cavity (*n* = 27), hypopharynx (*n* = 8), larynx (*n* = 7), orbita (*n* = 6), paranasal sinus (*n* = 2) and others (*n* = 7). A radial forearm flap was used for HNR in 109 cases, an anterolateral thigh flap in 9 cases, a latissimus-dorsi flap in 3 cases and a gracilis flap in 3 cases.

76 patients underwent HNR with EEA, 48 with ESA. Postoperative venous complications were observed in 5.3% (*n* = 4) and 2.1% (*n* = 1), respectively. A flap loss occurred in two cases after EEA (2.7%) and in one case after ESA (2.1%). Postoperative arterial complications occurred in two cases in the EEA group (one flap loss) but not in the ESA group.

The duration of anastomosis was measured in 20 cases in each group. The average EEA time was 4:16 min (SD 44 s), the average ESA time was 6:04 min (SD 93 s), *p* < 0.05 (student’s *t* test).

## Conclusion

The microvascular anastomosis remains one of the most technically challenging aspects of free tissue transfer reconstructions. Failure can lead to flap necrosis with consecutive severe healing problems, fistula formation and even life-threatening complications. The anastomotic coupling device has been shown to allow for success rates comparable or even superior to those seen with sutured anastomoses. In addition, the technique allows a reduction in the duration of surgery and makes it possible to compensate for diameter mismatches [5–8]. Unlike hand-sewn anastomoses, the use of the coupler allows eversion of the vessel walls by 90°, thus allowing both vessel lumens to be viewed. This significantly reduces the risk of exposure of the highly thrombogenic adventitia to the lumen, uneven placement of sutures or unrecognized backwalling of a stich. So far, microvascular coupling devices have mainly been accepted for use in end-to-end venous anastomoses [9, 10]. Only few authors have reported its use for ESA [5, 7, 11].

Here, it is demonstrated that ESA is a valuable alternative to EEA when using a coupler device providing more flexibility to the surgeon especially when it comes to HNR in patients with difficult anatomy, for example, after radical neck dissection or in cases of salvage surgery when the choice of recipient vessels is limited. The IJV is a constant, large and usually available vessel (at least on one side of the neck) and allows anastomoses of any size and number and at any location along the neck. Furthermore, the surgeon does not have to worry about different vessel diameters as ESA using the coupling device allows anastomosis of vessels with significant size discrepancy. The size of the coupler is dictated by the diameter of the flap vein. It is recommended to use the largest possible coupler size that fits the flap vein, as this significantly reduces the revision rate [4].

In both patient groups, the complication rate was rather low and comparable to the literature [5, 11]. Although some authors advocate EEA because of laminar flow ESA does not increase the incidence of anastomotic failure, according to the literature [4, 5].

We observed a significant difference in the duration of the anastomosis, which was longer in the ESA group. This might be due to the fact that the vessel wall of the internal jugular vein is thicker and a bit more difficult to handle than that of smaller veins such as the facial vein. With an average time of slightly more than 6 min ESA using a coupler is a lot less time-consuming than hand-sewn ESA which take around 25 min [1, 5–7, 9–12]. Further advantages of coupler anastomoses are technical ease of usage and a shorter learning curve.

## Supplementary Information

Below is the link to the electronic supplementary material.

Video 1: The IJV is shirred by pulling the upper and lower sections of the vein toward each other with two forceps before a Satinsky clamp is applied. (MP4 47316 KB)

Video 2: The size of the venotomy is controlled using a measuring gauge. (MP4 62923 KB)

Video 3: The flap vein is mounted on the steel pins of the coupler ring. (MP4 56213 KB)

Video 4: Flushing with heparin solution. (MP4 13729 KB)

Video 5: The IJV is mounted on the steel pins of the second coupler ring. (MP4 37802 KB)

Video 6: Closure of the coupler device. (MP4 55384 KB)

Video 7: The rings are gently pressed together to make sure that they interlock. (MP4 33835 KB)

Video 8: Donor and recipient vein are reopened and fill with blood. (MP4 21612 KB)

Video 9: The diameter of the IJV is slightly reduced at the site of the anastomosis. (MP4 15302 KB)

## Data Availability

Not applicable.
